# Supplementary material for: Genital GVHD in Female Children and Adolescents: A Systematic Review of Case Reports and Case Series
Source: Children (Basel). 2023 Aug 28;10(9):1463. doi: 10.3390/children10091463 (PMC10527655; doi:10.3390/children10091463)
Supplement: Supplementary file 1 [file children-10-01463-s001.zip › children-2482689-supplementary.pdf]

Supplementary Table S1. Risk of Bias Assessment using NIH Quality Assessment Tool for Case Series Studies

Study	Dowlut-McElroy 2022	Cicek 2019	Michala 2018	Allen 2020	Choi 2009	Stratton 2007	Childress 2015
1. Was the study question or objective clearly stated?	Yes	Yes	Yes	Yes	Yes	Yes	Yes
2. Was the study population clearly and fully described, including a case definition?	Yes	Yes	Yes	Yes	Yes	Yes	No
3. Were the cases consecutive?	Yes	Yes	N/A	Yes	N/A	Unclear	N/A
4. Were the subjects comparable?	Yes	Yes	Yes	Yes	Yes	Yes	Yes
5. Was the intervention clearly described?	Yes	Yes	Yes	Yes	Yes	No	Yes
6. Were the outcome measures clearly defined, valid, reliable, and implemented consistently across all study participants?	Yes	Yes	Yes	Yes	Yes	No	Yes
7. Was the length of follow-up adequate?	Yes	Yes	Yes	Yes	Yes	No	Yes
8. Were the statistical methods well-described?	Yes	Yes	N/A	Yes	N/A	Yes	N/A
9. Were the results well-described?	Yes	Yes	Yes	Yes	Yes	Yes	Yes
